# Design Optimization and Characterization with Fabrication of Nanomaterials-Based Photo Diode Cell for Subretinal Implant Application

**DOI:** 10.3390/nano13050934

**Published:** 2023-03-04

**Authors:** Vijai M. Moorthy, Joseph D. Rathnasami, Viranjay M. Srivastava

**Affiliations:** 1 Department of Electronic Engineering, Howard College, University of KwaZulu-Natal, Durban 4041, South Africa; 2 Department of Electronics and Instrumentation Engineering, Faculty of Engineering and Technology, Annamalai University, Chidambaram 608 002, India

**Keywords:** flexible substrate, graphene, subretinal prostheses, nanomaterials, carbon nano tube, microelectronics, nanotechnology

## Abstract

An ultrathin nano photodiode array fabricated in a flexible substrate can be an ideal therapeutic replacement for degenerated photoreceptor cells damaged by Age-related Macula Degeneration (AMD) and Retinitis Pigmentosa (RP), such as retinal infections. Silicon-based photodiode arrays have been attempted as artificial retinas. Considering the difficulties caused by hard silicon subretinal implants, researchers have diverted their attention towards organic photovoltaic cells-based subretinal implants. Indium-Tin Oxide (ITO) has been a favorite choice as an anode electrode. A mix of poly(3-hexylthiophene) and [6,6]-phenyl C61-butyric acid methyleste (P3HT: PCBM) has been utilized as an active layer in such nanomaterial-based subretinal implants. Though encouraging results have been obtained during the trial of such retinal implants, the need to replace ITO with a suitable transparent conductive electrode will be a suitable substitute. Further, conjugated polymers have been used as active layers in such photodiodes and have shown delamination in the retinal space over time despite their biocompatibility. This research attempted to fabricate and characterize Bulk Hetero Junction (BHJ) based Nano Photo Diode (NPD) utilizing Graphene–polyethylene terephthalate (G–PET)/semiconducting Single-Wall Carbon Nano Tubes (s-SWCNT): fullerene (C_60_) blend/aluminium (Al) structure to determine the issues in the development of subretinal prosthesis. An effective design approach adopted in this analysis has resulted in developing an NPD with an Efficiency of 10.1% in a non-ITO-driven NPD structure. Additionally, the results show that the efficiency can be further improved by increasing active layer thickness.

## 1. Introduction

The retina in the eye transforms the light information into neural electrical impulses that the optic nerve transfers to the brain’s visual cortex. The visual cortex decodes the neural impulses into meaningful image perception. Various neural cell layers, such as photoreceptor cells, amacrine cells, bipolar cells, and axon ganglion cells, are responsible for signal processing and convergence [1].

Age-related Macula Degeneration (AMD) and Retinitis Pigmentosa (RP) are the two retinal disorders that affect an increasing number of people worldwide. Over 55-year-olds are most likely to be affected by AMD, which destroys the macula, a region of about 5 mm in diameter that surrounds the retina center [2]. A tiny pit termed a fovea with a diameter of around 1.5 mm is located in the macula center, where cones are abundant (10%). It is responsible for the sharpest vision. Therefore, people with AMD have central vision loss. Retinitis pigmentosa (RP) is a genetic illness characterized by outer retinal degeneration [3]. As a result, photoreceptors’ outer and inner segments and their cell bodies are damaged.

There is currently no effective therapy for AMD or RP. Conversely, even after periods of loss of sight, around 30% of retinal ganglion cells in RP patients remain functional [4]. Epi-retinal implant and Sub-retinal implant are two techniques that have been used to describe retinal prosthesis during the past two decades.

Epi-retinal implants employ electrical stimulation to produce visual perception with fewer electrodes, and all image processing is performed outside, similar to ARGUS II [5]. Although Argus^®^ II’s epiretinal implantation technique successfully produces phosphenes in response to visual stimulus [4], as a result of image persistence, the apparent image may fade in a few seconds. It is too complicated to continue the surgical procedure as it requires external instruments. Hence, the epiretinal approach is not widely adopted. To function, the sub-retinal implant replaces deteriorated photoreceptors with stimulating photodiode electrodes and processes the images internally, similar to the Silicon Micro Photo Diode Array (SMPDA), Alpha IMS, which is the most widely researched approach because of its various advantages [4]. For example, single-letter discrimination and rudimentary object recognition were proven in the preliminary studies on Alpha IMS [6]. According to clinical trials on such prostheses, an array of 25 × 25 pixels and a 30-degree viewing angle might give appropriate mobility skills [7,8].

Chow et al. [9,10,11,12,13] designed an SMPDA for a subretinal prosthesis (Artificial Silicon Retina (ASR), Optobionics Corp.) with the belief that a subretinal implant could be powered solely by ambient light. As a replacement for the degenerated photoreceptors, this SMPDA was placed between the pigment epithelium of the retina (RPE) and the bipolar cell layer. The results showed improvements in visual perception. A consortium in Germany coordinated by E. Zrenner [14,15,16,17,18,19,20] has been developing amorphous-based silicon photodiodes for subretinal implantation. This is the first time a thin film MPDA has been implanted into the eyes of rats and rabbits for early testing. In this device (a-Si: H/Ti), the higher intensity of infrared light is locally enhanced to boost the photo response. Many contact layers have been studied to offer strong perpendicular conductivity with less lateral parasitic losses to the adjacent tissue due to the need for optimal capacitive coupling to bipolar cells in activating retinal cells. In vitro results have elucidated that small photovoltaic cells (100 µm × 100 µm and 20 µm × 20 µm) cannot provide sufficient charge capacitive to stimulate the bipolar cells. To this end, huge (200 µm × 200 µm) solar cells have been constructed to generate two-volt potentials. However, in vivo experimental results showed that the SiO_2_-coated passivation layer device is corrosive after six months. Later the same group [14,15,16,17,18,19,20] developed a new MPDA prototype with discrete materials using a polyimide foil that is both flexible and strong, incorporating an MPDA chip with stimulation electrodes, a controller circuit, and an IR receiver module for energy transmission. By implantation of their prosthesis in animals, they investigated different parameters such as successful electrical stimulation, durable biocompatibility, and stability of the subretinal implants. There were tiny dislocations of the subretinal portions of the implant in animals, which eventually led to a tear in the retina. Techniques were used to ensure the subretinal stimulation components were positioned correctly. The devices implanted after the modified procedure on seven RP patients showed startling improvements in visual function despite surgical complications. This MPDA created a detailed, meaningful visual perception in already blind individuals permitting localization and recognition of objects up to perusing ability.

Further, the implanted MPDA showed that it was both safe and productive. Later, Zrenner et al. [21] developed an improved version of MPDA with 1500 photosensitive pixels, each with its photodiode, stimulation electrode and amplifier, and a grid of 16 wire-associated electrodes that enables light-independent direct stimulation to take place. Their clinical pilot study demonstrated that the subretinal implant gives meaningful visual information to blind patients [22].

Using photovoltaic stimulation, according to Palanker et al. [23,24,25], a unique idea was developed in 2012 that overcomes present constraints, such as mechanical mismatch with delicate retinal tissues and the complexity of manufacturing process and operating principles [21,22]. There is no need for implants or physical connections with external devices when infrared light enters the pupil and is transformed into electrical impulses that are supplied to the retina in this configuration. However, the main problem with such a method is its poor sensitivity, which makes it difficult to use under daytime conditions [23,24,25]. In addition to improving visual acuity, expanding the field of vision beyond blindness thresholds, and overcoming medical complications, the requirement for trans-ocular wires to deliver power and control signals, as well as corrosion in the implants after a few months, significantly impairs their functionality; there are still many obstacles to overcome. This was because most MPDAs were built on hard silicon substrates; their stiffness damages the retinal tissues. It is also difficult to integrate artificial functional materials with live neural tissues [26,27,28]. Therefore, the design of a sub-retinal implant device based on a foldable substrate material without any external discrete components was deemed urgent.

Recently, photodiodes using organic and nanomaterials on foldable substrates have been widely reported. The ease with which organic electronics and biological substrates may be interfaced opens new research opportunities [29,30]. Light-induced retinal stimulation via semiconductor nanorod–carbon nanotube implants was created by Bareket et al. [31] using Poly-Di-Methyl-Siloxane (PDMS) substrate material. The results suggested the possible use of this device in future retinal applications. Some recent analysis on organic photovoltaic devices has been conducted for neuronal photostimulation [32,33]. This technology offers significant advantages over inorganic semiconductors and has considerable promise; according to the researchers, the material is suitable for in vivo biological applications such as retinal prostheses. A subretinal organic photovoltaic interface was developed by Ghezzi et al. [32,33] by combining organic semiconductors and conjugated polymers (i.e., poly(3,4-ethylene-dioxythiophene)-poly (styrenesulfonate, PEDOT: PSS; regioregular poly (3-hexylthiophene-2,5-dial), P3HT; [6,6]-phenyl-C61butyric acid methyl ester, PCBM).

Photovoltaics, mechanical compliance, and daylight sensitivity drive conjugated polymers, unlike silicon-based prostheses. Ghezzi et al. [34,35,36] improved dystrophic rats’ visual acuity, but issues persisted one month after implantation. Subretinal conjugated polymers delaminate in months, destroying organic molecules irreversibly. Neural photostimulation may result from polymer–electrolyte capacitive [34,35,36]. In 2015, Narayan et al. [37] constructed an organic-based artificial retina device employing a photoconductive polymer blend, such as poly (3,4-ethylene dioxythiophene): poly (4-styrene sulfonate) (PEDOT: PSS). Silicon or organic photovoltaic subretinal implants are too tiny to improve vision [38], such as in the case of risky subretinal implants (for example, retinal peeling, device movement, and device overlapping). Retinal prosthetics’ biggest obstacles are vision and field size [39], as with organic conjugated polymer retinal prosthesis [34,35,36,37] targeting the active layer.

ITO decreases Voc. Conductive graphene electrodes replace ITO in organic photovoltaics [40,41,42]. ITO-electrodes C60–SWCNT active layer photovoltaic cells worked well [43]. s-SWCNTs outperform conjugated organic polymers in UV/visible photovoltaics [44,45]. C60–SWCNT composites on graphene transparent electrodes are superior to ITO/C60-SWCNT/Al [46]. In this work, the present authors fabricated and characterized nano photodiodes that use BHJ formed with a G-PET/S-SWCNT: C_60_/Al structure for the subretinal implant to completely avoid the usage of ITO. Further, the SWCNT: C_60_ active layer replaces P3HT: PCBM to enhance the performance of subretinal prosthesis [33]. This article proposes a subretinal prosthesis design utilizing nanomaterials that include polymer, carbon nanotubes, graphene, and aluminum. In addition, this work focuses on the fabrication and characterization of a high-performance single nano photodiode. The primary objective is to effectively design and construct an NPD that can provide the appropriate current and voltage output, which is very important for the implant due to the retina prosthesis’s current mode and voltage mode. The structure of this article is as follows: The structure of the BHJ nano photodiode is discussed in Section 2, and the fabrication process flow is discussed in Section 3. The standardization of processes and the optimization of device layers are then discussed in Section 4. Fabrication characteristics, findings, and discussion are covered in Section 5; in addition, Section 6 draws comparisons to previously existing NPD PV cells in terms of the results and analysis. Finally, Section 7 concludes the work and recommends future approaches.

## 2. Structure of BHJ Nano Photo Diodes

Solar cells use the photovoltaic effect to convert light into energy, a process known as photovoltaics or photovoltaic conversion. Pure energy, light, enters a PV cell and provides a few electrons (negatively charged atomic particles) with enough energy to release them from their entanglement. On the other hand, photovoltage can be generated via a built-in potential barrier, which acts on these electrons to form a voltage.

Organic photovoltaics utilize organic semiconductors to absorb light and produce electricity. Organic semiconductors are carbon-based materials that possess semiconductor properties. The donor and acceptor phases are separated in a bilayer heterojunction [47]. The donor and acceptor materials are extensively blended in a Bulk Hetero Junction (BHJ) type to decrease the distance of the interface areas from the point of exciton generation [47], allowing the exciton to travel much less than the diffusion length to avoid recombination. While the bilayer structure has a 2D donor-acceptor interface in the BHJ device, this interface is three-dimensional. Because of this, the interface area in BHJ is enlarged by many orders of magnitude, and exciton dissociation is considerably more efficient than in BHJ. For better light absorption, the active layer’s thickness might be selected to be much greater than the exciton diffusion length.

The proposed NPD structure includes a polymer, carbon nanotubes, graphene, and aluminum. This NPD generates power under incident light to stimulate the retina for regaining lost vision function. After depositing an electrode and a mix of donor and acceptor materials in a bulk volume, a junction between two distinct materials is formed. Bulk Hetero Junction solar cell is the name given to this type of cell.

**Figure 1:**
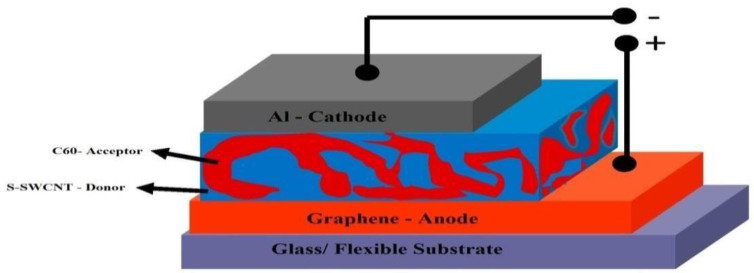
Structure of Bulk Hetero Junction (BHJ) Single Nano Photo Diode cell.

Figure 1 shows the suggested structure of a nano photodiode. Semiconducting Single-Wall Carbon Nano Tubes (S-SWCNT) are used as electron donors, C_60_ fullerene is used as an electron acceptor, and graphene is used as an anode in this experiment. The efficiency and transparency of the NPD may be enhanced; the s-SWCNT:C_60_ were mixed in the ratio of (1:100), the chirality of the SWCNT is (6,5), the purpose of utilizing these active layer nanomaterials is that they provide efficiency, stability, are good absorbers, and provide better efficiency in near-infrared regions, these materials are treated on a flexible substrate [48,49]. Transparency plays an important role in organic photovoltaic devices. If the electrode transparency is high, then optical transmittance is maximized while organic polymers absorb ultraviolet (UV) and Near-Infra Red (NIR) radiation.

Figure 1 shows a structure that blends two semiconducting materials, a donor (SWCNT) and an acceptor (C_60_ fullerene), with distinct band gaps positioned between two electrodes (graphene, 5.3 eV anode, and aluminum, 4.2 eV cathode), to form a junction that aids in charge separation of excitons [43,46,47,48,49,50].

These two layers, graphene and aluminum electrodes, create exciton, diffusion of excitons, and free carriers. We initially used aluminum as a cathode material since it is one of the most popular cathode materials in organic photovoltaics. Ideally, the cathode should be made of metal with a lower work function than the anode. The work function of graphene is greater than aluminum, thus meeting the criteria.

### 2.1. Device Physics of Bulk Heterojunction Photovoltaic Cells

In BHJ, the donor and acceptor materials are mixed in large quantities Figure 1. Donor–acceptor phase separation may be seen across length scales of 10–20 nm. Since every interface is inside the exciton diffusion length of the absorber, the system is interpenetrating at the nanoscale [51].

### 2.2. Characteristics of OPV

**Figure 2:**
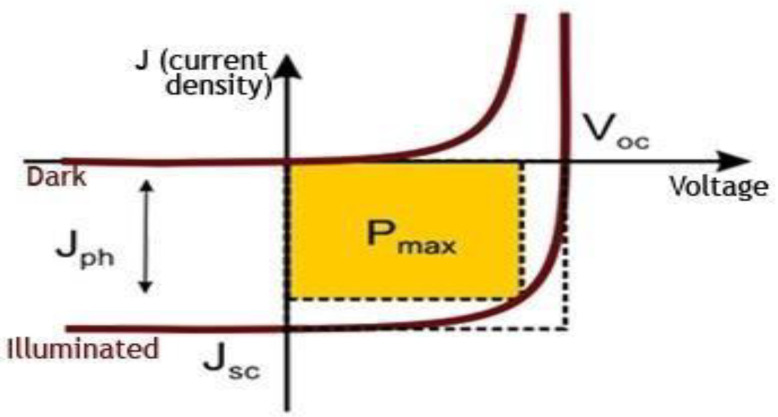
Typical (I-V) curves for an Organic Photo Voltaic (OPV) (dark and illuminated).

Figure 2 shows the current-voltage curves of a BHJ Organic photovoltaic cell in the dark and the light. There are four essential characteristics used to describe solar cells: open circuit voltage (V_oc_), short circuit current (J_sc_), efficiency, and Fill Factor (FF), which are obtained using the following formula:

(1) $$J_{sc}=qG(L_{n}+L_{p})$$

where G is the generation rate, L_n_ and L_p_ are the electron and holes transmission length, respectively.

(2) $$V_{oc}=\frac{nKT}{q}\ln\left(\frac{I_{L}}{I_{o}}+1\right)$$

where I_o_ and I_L_ are the dark saturation current and light generated current, respectively, n is ideality factor, kT/q is thermal voltage and P_in_ is input power, and E is the incident radiation.

(3) $$FF=\frac{P_{\max}}{J_{sc}\times V_{oc}}$$

(4) $$\eta =\left|\frac{P_{\max}}{E}\right|\times 100\%$$

where I_max_, and V_max_ are the operating points of maximum power for current and maximum voltage, respectively.).

## 3. Fabrication Process Flow of NPD Device

This section describes the BHJ-based Nano Photo Diode (NPD) fabrication method used in this work. The structure of the BHJ OPV cell is represented as Graphene/S-SWCNT: C_60_ blend/Al. It is realized using spin coating and shadow masking techniques.

### Fabrication Steps

There are several steps involved in the fabrication that takes it from substrates to devices ready for measurement.

*a*. *Anode Layer Coating*

Graphene is picked as an anode material. Here, the authors utilized a graphene-coated PET with a thickness of 50 nm.

*b*. *Formation of Photo Active Layer*

**Figure 3:**
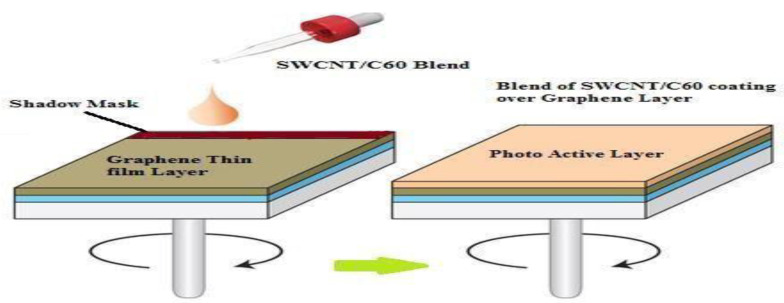
Spin coating of Active layer over graphene thin film Layer.

The present authors used a blend of S-SWCNT: C_60_ as an active layer. In this work, the active layer mix proportions and fixations have been chosen from previously optimized conditions [46]. Using 1,2 Dichlorobenzene, S-SWCNT was dissolved to a concentration of 1 mg/mL. Similarly, dissolving 100 mg of C_60_ in 100 mL of 1,2-dichlorobenzene is then ultrasonically heated at 20 °C in a glove box for 2 h. The following is the process for preparing this blend: as a final blend, combine the two solutions produced as mentioned above at a concentration of 1% S-SWCNT. A small area of the graphene sheet must be covered with adhesive tape before applying the mix (Keaton tape). In the spin coating deposition technique, it is termed shadow masking. To characterize the device, the protected area is utilized for contact purposes. The mix is then spin-coated over the graphene-coated PET substrate, as shown in Figure 3 [52].

*c*. *Cathode Layer Deposition*

For the device to function, thermal evaporation at a vacuum more significant than 2 × 10–26 torr was used to deposit an Al cathode layer over the active layer. As a result, the shadow mask must be properly positioned over the coated substrate to prevent aluminium deposition over the graphene layer. Here, the al layer is deposited by covering some portions using aluminium foil, and the Al is deposited in the uncovered region. The thickness of the aluminium layer coated is approximately 50 nm.

## 4. Optimization Design of Device Layers and Process Standardization

The NPD developed in this work is ultimately used for subretinal implantation. Therefore, such NPD should have the capability to provide large charge output even for lower optical power. To achieve this, the device should be highly efficient in converting light energy into electrical power. This will mainly depend upon the design and optimization of various layer thicknesses and their electrical behavior [53,54]. Therefore, before the devices were fabricated, the authors performed optimizations of various layer thicknesses.

### 4.1. Active Layer Thickness Optimization

In this work, the S-SWCNT: C_60_ blend is used as an active layer. Now, the thickness of this active layer for maximum efficiency must be determined. The authors of this present work conducted an optical modeling of the device to investigate the effect of the influence of the optical constants and active layer thickness of the blend films on the performance of the solar cells. Based on transfer matrix formalism, an empirical optoelectronic model was used to properly predict the photocurrent in Organic Photovoltaic (OPV) devices comprising BHJ OPV cells at varied active layer depths. In addition, parasitic absorption is considered at the electrodes [55]. Our simulations for varying film thicknesses in the photoactive layer used the optical constants of all layers in the examined device structure (up to 150 nm). The thickness of the graphene and aluminum layers was 50 and 100 nanometers, respectively. Afterward, compute the device’s V_oc_ and J_sc_.

Here, the authors have simulated four polymer solar cells [56] (G-PET/s-SWCNT: C_60_/Al), varying the blend film thickness throughout the 50–150 nm range, as shown in Table 1. The simulation findings show unequivocally that increasing the active layer thickness improves the performance of the BHJ device. Hence, it is important to realize a film thickness of 150 nm in the range when the blend of S-SWCNT: C_60_ is coated.

### 4.2. Cathode Layer Thickness Optimization

In the BHJ photovoltaic cell’s cathode layer, aluminum is used, and the influence of aluminum film thickness on the cell’s performance was also examined using the same modeling tool. The anode and active layer thickness are set to 50 nm and 100 nm, respectively. Table 2 shows that the thickness of the cathode layer does not affect the solar cell performance. In a solar cell, the cathode is located at the bottom [56]. As a result, it does not affect optical absorption. To collect charges, the cathode must be used. A thin cathode film will not fully collect charges that arrive at the cathode; thereby, it may reduce efficiency. Thick cathode thickness will fully collect charges that arrive at the cathode, but a large thickness is undesirable. However, the results show that the Al layer thickness has no significant role if the thickness lies between 10 nm and 125 nm.

## 5. Fabrication Characterization, Results, and Discussion

The BHJ PV cell simulations demonstrate that the active layer should be as thick as possible to maximize efficiency. To ascertain this, we fabricated four devices A, B, C, and D. These devices were fabricated with active layers S-SWCNT: C_60_ coated at spin speeds of 1200, 1000, 800, and 600 rpm, respectively [44], as presented in Table 3. The cathode layer thickness is fixed to be 50 nm. The devices, A, B, C, and D are realized using the fabrication process described in Section 3.

The current voltages in the dark and under simulated AM 1.5G light (100/cm^2^) were then measured using a solar simulator Keithley 2400 Source meter. The results obtained are summarized in Table 4. The Jsc is 19.05/cm^2^, the voltage was 0.75 V, the Fill factor (FF) was 79.56%, and PCE was 10.1% for device D. According to the comparison of these results with the modeling results presented in Table 1, device D must have an active layer thickness of about 60 nm. A comparison of Table 3 and Table 4 further shows that the decrease in spin speed will increase the PCE.

Table 4 indicates that the device performance increased from 3.85% to 10.1% because of the graphene substrate. In our previous work, we utilized the graphene coating over the glass substrate, and the surface uniformity was very poor; hence, in this present work, we utilized a precoated graphene substrate, G-PET, to overcome the drawbacks in the previous work. The results also clearly indicate that the device performance is increased.

## 6. Comparison with Reported NPD PV Cells

The anode in an OPV cell should be a good conductor of electricity and, simultaneously, must be highly transparent to light. This makes the selection of an anode a difficult task. ITO/PEDOT: PSS transparent electrode has been extensively used as a transparent anode electrode. However, ITO-based anode electrode suffers many disadvantages, as discussed in the introduction section, and the researchers have been looking for better material. Nowadays, ITO has been replaced by Graphene/PEDOT: PSS and P3HT: PCBM as active materials. In this work, the authors directed their effort to optimize graphene sheets’ transparency and electrical conductivity so that graphene alone acts as the anode electrode and the fabrication process becomes easy while still providing matching or better performance with ITO-based OPV cells. The active layer has also been changed to SWCNT: C_60_ blend, considering the difficulties in using P3HT: PCBM in subretinal implants [32,33,34,35,36]. Now to bring out the effectiveness of this work, a summary of PCEs of the OPV devices employing ITO/PEDOT: PSS transparent electrode and conventional P3HT: PCBM as active materials is presented in Table 5. The performance of OPV cells that employ graphene-based films for anode layers but with conventional P3HT: PCBM or SWCNT: C_60_ as active layer are listed in Table 6. It is clear from Table 5 results that ITO/PEDOT: PSS-based devices can provide an efficiency of 3.86% with P3HT: PCBM as active material. When PEDOT forms the anode: PSS alone, the efficiency drastically falls, indicating that the ITO provides the transparency required for optical transmission.

Similarly, the reported results in Table 6 show that Graphene/PEDOT: PSS can be an ideal choice in place of ITO/PEDOT: PSS in the OPV cells using P3HT: PCBM as an active layer. A PCE of 3.98 was obtained as a maximum [64]. The results obtained in this analysis demonstrated that Graphene/PEDOT: PSS could be replaced with a single graphene layer anode electrode to improve the OPV cell’s efficiency, employing the S-SWCNT/C_60_ blend as an active layer.

The other main difference in our work is the active layer material. A blend of SWCNT: C_60_. An SWCNT donor and C_60_ acceptor material are utilized instead of the typical P3HT: PCBM active material. In comparison, with previously reported graphene and ITO-based conjugated polymer devices shown in Table 5 and Table 6 [57,58,59,60,61,62,63,64,65,66,67,68], our devices with G-PET/S-SWCNT: C_60_/Al structure show better performance with a PCE of 10.1%, J_sc_ = 19.05 mA/cm^2^, FF = 79.56, and V_oc_ = 0.75 V. It is possible to improve this efficiency further if the active layer is spun at lower speeds (<600 rpm) as it is evident from Table 1, Table 3 and Table 4. Further, it is essential to note that the present work has achieved the best PCE so far with S-SWCNT/C_60_ employed OPV cells, as evidenced by Table 6 and Table 7.

## 7. Conclusions and Future Recommendation

Recently, photodiodes using organic and nanomaterials on foldable substrates have been widely reported for prostheses. The ease with which organic electronics may be interfaced with biological substrates opens new possibilities for artificial prosthesis applications. In this work, we fabricated and characterized nano photodiodes that use the G-PET/S-SWCNT: C_60_/Al BHJ structure for a subretinal implant. An effective design approach is adopted in this analysis that resulted in developing an NPD with an Efficiency of 10.1%. Additionally, the obtained results show that the device performance is much superior when compared to the previously reported works. This will help to increase the number of electrodes when aiming for the fabrication of nano photodiode arrays for a subretinal implant that will help to increase the visual acuity and visual field of the device. The main advantage of the fabricated NPD is its materials, the materials utilized in the device will work under the near-infrared region.

Further simulation analysis brought out the effect of cathode layer thickness on PCE. ITO has been replaced with graphene as a transparent electrode because of its high electron and hole mobility. Further, the SWCNT: C_60_ active layer substitutes P3HT: PCBM to improve the effectiveness of the subretinal prosthesis. The test results demonstrated that our device performed better than other such reports. Additionally, the results indicate that the PCE can be increased with suitable modification in active layer thicknesses.

In the future, this NPD device will be realized in a flexible substrate with an array of electrodes and tested in biological environments.

## Figures and Tables

**Table 1:** Simulation results for different active layer thickness.

Active Layer Thickness (nm)	V_OC_ (V)	Jsc (mA/cm^2^)	FF (%)	Power Conversion Efficiency (PCE) (%)
50	0.73	11.329	44.36	3.68
75	0.75	12.036	46.31	4.1
100	0.78	12.743	48.24	4.76
150	0.78	14.157	48.75	5.3

**Table 2:** Simulation results for different cathode layer thickness.

Al Thickness (nm)	Voc (V)	Jsc (mA/cm^2^)	FF (%)	PCE (%)
10	0.724	12.68	51	5.16
20	0.725	12.69	51	5.16
30	0.727	12.71	51	5.16
40	0.728	12.73	51	5.16
50	0.734	12.73	51	5.16
75	0.736	12.74	52	5.16
100	0.737	12.75	52	5.16
125	0.738	12.75	52	5.17

**Table 3:** Active layer coating with different spin speed.

Active Layer: (SWCNT: C60) Blend	
Device Name	Spin Speed (rpm)
A	1200
B	1000
C	800
D	600

**Table 4:** Comparison of NPD performance parameters for various devices.

Sample	Existing Work Using Graphene Coating (Over the Glass Substrate)				This Work, Graphene Coating in PET Substrate			
	VOC (V)	Jsc (mA/cm^2^)	FF (%)	PCE (%)	VOC (V)	Jsc (mA/cm^2^)	FF (%)	PCE (%)
A	0.58	4.276	48.5	1.20	0.68 (±0.00)	11.34 (±0.26)	70.00 (±0.45)	5.7 (±0.08)
B	0.63	6.21	50.0	1.96	0.69 (±0.00)	12.05 (±0.59)	71.05 (±1.02)	6.43 (±0.16)
C	0.62	10.55	49.2	3.20	0.71 (±0.00)	16.3 (±0.17)	73.00 (±0.45)	7.52 (±0.11)
D	0.68	12.13	46.6	3.85	0.75 (±0.00)	19.05 (±0.26)	79.56 (±0.45)	10.1 (±0.75)

**Table 5:** Summary of ITO-based electrodes with the best PCEs for OPV cells.

REF	Anode Layer	Active Layer	Cathode	PCE (%)
[57]	ITO/PEDOT: PSS	P3HT: PCBM	Al	0.55
[58]	ITO/PEDOT: PSS	P3HT: PCBM	Al	2.93
[59]	ITO/PEDOT: PSS	P3HT: PCBM	Al	4.37
[60]	ITO/PEDOT: PSS	P3HT: PCBM	LiF-Al	1.74
[60]	AgOx-ITO/PEDOT: PSS	P3HT: PCBM	LiF-Al	2.25
[61]	ITO/PEDOT:PSS+Au	P3HT: PCBM	Al	3.23
[62]	PEDOT: PSS	P3HT: PCBM	Al	0.0038
[63]	ITO/PEDOT: PSS	P3HT: PCBM	Al	3.56
[64]	ITO/PEDOT: PSS	P3HT: PCBM	Al	3.86

**Table 6:** Summary of graphene-based electrodes having the best PCEs employing them in OPV cells.

REF	Anode Layer	Active Layer	Cathode	PCE (%)
[65]	G-CNT/PEDOT: PSS	P3HT: PCBM	Al	0.85
[66]	rGO/PEDOT: PSS	P3HT: PCBM	TiO2/Al	0.77
[63]	Stacked Graphene/PEDOT: PSS	P3HT: PCBM	Ca/Al	0.89
[64]	Graphene/PEDOT: PSS	P3HT: PCBM	Al	3.98
[65]	Graphene	SWCNT: C_60_	Al	3.85
This work	G-PET	SWCNT: C_60_	Al	10.1

**Table 7:** Summary of S-SWCNT-based active layer having best PCEs employing them in OPV cells.

REF	Anode Layer	Active Layer	Cathode	PCE (%)
[43]	ITO	P3HT: C_60_–SWCNT	Al	0.57
[43]	ITO	P3HT: C_60_	Al	0.38
[67]	ITO/PEDOT: PSS	P3HT: SWCNT: PCBM	Al	2.38
[45]	ITO/PEDOT: PSS	s-SWCNT/PC71BM	BCP/Al	2.5
[45]	ITO/PEDOT: PSS	s-SWCNT/PC71BM	Moox/Ag	3.1
[46]	Graphene	SWCNT: C_60_	Al	0.81
[65]	Graphene	SWCNT: C_60_	Al	3.85
This work	G-PET	SWCNT: C_60_	Al	10.1

## Data Availability

All data and materials used to prepare this manuscript are available in this document.

## References

[1] Meyer J.U. (2002). Retinal Implanta bioMEMS Challenge. Sens. Actuators A: Phys..

[2] van Lookeren Campagne M., LeCouter J., Yaspan B.L., Ye W. (2014). Mechanisms of age-related macular degeneration and therapeutic opportunities. J. Pathol..

[3] Flannery J., Farber D., Bird A.C., Bok D. (1989). Degenerative Changes in a Retina Affected with Autosomal Dominant Retinitis Pigmentosa. Investig. Opthalmology Vis. Sci..

[4] Mokwa W. (2008). 3.06 Artificial Retinas. https://cpb-us-w2.wpmucdn.com/blog.nus.edu.sg/dist/a/315/files/2010/03/3.06-Artificial-Retinas.pdf.

[5] Luo Y.H.-L., da Cruz L. (2016). The Argus® II Retinal Prosthesis System. Prog. Retin. Eye Res..

[6] Stingl K., Bartz-Schmidt K.U., Besch D., Chee C.K., Cottriall C.L., Gekeler F., Groppe M., Jackson T.L., MacLaren R.E., Koitschev A. (2015). Subretinal Visual Implant Alpha IMS—Clinical trial interim report. Vis. Res..

[7] Cha K., Horch K.W., Normann R.A. (1992). Mobility Performance with a Pixelized Vision System. Vis. Res..

[8] Dagnelie G., Keane P., Narla V., Yang L., Weiland J., Humayun M. (2007). Real and virtual mobility performance in simulated prosthetic vision. J. Neural Eng..

[9] Chow A.Y., Peachey N.S. (1998). The subretinal microphotodiode array retinal prosthesis. Ophthalmic Res..

[10] Peyman G., Chow A.Y., Liang C., Chow V.Y., Perlman J.I., Peachey N.S. (1998). Subretinal semiconductor micro photodiode array. Ophthalmic Surg. Lasers Imaging Retin..

[11] Chow A.Y., Peachey N. (1999). The Subretinal Microphotodiode Array Retinal Prosthesis II. Ophthalmic Res..

[12] Chow A.Y., Pardue M.T., I Perlman J., Ball S.L., Chow V.Y., Hetling J.R., A Peyman G., Liang C., Stubbs E.B., Peachey N.S. (2002). Subretinal implantation of semiconductor-based photodiodes: Durability of novel implant designs. J. Rehabilitation Res. Dev..

[13] Chow A.Y., Chow V.Y., Packo K.H., Pollack J.S., Peyman G.A., Schuchard R. (2004). The Artificial Silicon Retina Microchip for the Treatment of Vision Loss from Retinitis Pigmentosa. Arch. Ophthalmol..

[14] Faber H., Ernemann U., Sachs H., Gekeler F., Danz S., Koitschev A., Besch D., Bartz-Schmidt K.-U., Zrenner E., Stingl K. (2021). CT Assessment of Intraorbital Cable Movement of Electronic Subretinal Prosthesis in Three Different Surgical Approaches. Transl. Vis. Sci. Technol..

[15] Kapetanovic J.C., Troelenberg N., Edwards T.L., Xue K., Ramsden J.D., Stett A., Zrenner E., MacLaren R.E. (2020). Highest reported visual acuity after electronic retinal implantation. Acta Ophthalmol..

[16] Schwahn H.N., Gekeler F., Kohler K., Kobuch K., Sachs H.G., Schulmeyer F., Jakob W., Gabel V.-P., Zrenner E. (2001). Studies on the feasibility of a subretinal visual prosthesis: Data from Yucatan micropig and rabbit. Graefe’s Arch. Clin. Exp. Ophthalmol..

[17] Zrenner E., Gekeler F., Gabel V., Graf H., Graf M., Guenther E., Haemmerle H., Hoefflinger B., Kobuch K., Kohler K. (2001). Subretinales Mikrophotodioden- Array als Ersatz für degenerierte Photorezeptoren?. Der Ophthalmol..

[18] Cehajic-Kapetanovic J., Singh M.S., Zrenner E., MacLaren R.E. (2022). Bioengineering strategies for restoring vision. Nat. Biomed. Eng..

[19] Gekeler F., Szurman P., Grisanti S., Weiler U., Claus R., Greiner T.-O., Völker M., Kohler K., Zrenner E., Bartz-Schmidt K.U. (2006). Compound subretinal prostheses with extra-ocular parts designed for human trials: Successful long-term implantation in pigs. Graefe’s Arch. Clin. Exp. Ophthalmol..

[20] Besch D., Sachs H., Szurman P., Gulicher D., Wilke R., Reinert S., Zrenner E., Bartz-Schmidt K.U., Gekeler F. (2008). Extraocular surgery for implantation of an active subretinal visual prosthesis with external connections: Feasibility and outcome in seven patients. Br. J. Ophthalmol..

[21] Zrenner E., Bartz-Schmidt K.U., Benav H., Besch D., Bruckmann A., Gabel V.-P., Gekeler F., Greppmaier U., Harscher A., Kibbel S. (2010). Subretinal electronic chips allow blind patients to read letters and combine them to words. Proc. R. Soc. B: Boil. Sci..

[22] Wilke R., Gabel V.-P., Sachs H., Schmidt K.-U.B., Gekeler F., Besch D., Szurman P., Stett A., Wilhelm B., Peters T. (2011). Spatial Resolution and Perception of Patterns Mediated by a Subretinal 16-Electrode Array in Patients Blinded by Hereditary Retinal Dystrophies. Opthalmology Vis. Sci..

[23] Huang T.W., I Kamins T., Chen Z.C., Wang B.-Y., Bhuckory M., Galambos L., Ho E., Ling T., Afshar S., Shin A. (2021). Vertical-junction photodiodes for smaller pixels in retinal prostheses. J. Neural Eng..

[24] Mandel Y., Goetz G., Lavinsky D., Huie P., Mathieson K., Wang L., Kamins T., Galambos L., Manivanh R., Harris J. (2013). Cortical responses elicited by photovoltaic subretinal prostheses exhibit similarities to visually evoked potentials. Nat. Commun..

[25] Chen Z.C., Wang B.Y., Goldstein A.K., Butt E., Mathieson K., Palanker D. (2022). Photovoltaic implant simulator reveals resolution limits in subretinal prosthesis. J. Neural Eng..

[26] Wallace G.G., Moulton S.E., Clark G.M. (2009). Electrode-Cellular Interface. Science.

[27] Kotov N.A., Winter J.O., Clements I.P., Jan E., Timko B.P., Campidelli S., Pathak S., Mazzatenta A., Lieber C.M., Prato M. (2009). Nanomaterials for Neural Interfaces. Adv. Mater..

[28] Pappas T.C., Wickramanyake W.M.S., Jan E., Motamedi M., Brodwick M., Kotov N.A. (2006). Nanoscale Engineering of a Cellular Interface with Semiconductor Nanoparticle Films for Photoelectric Stimulation of Neurons. Nano Lett..

[29] Moorthy V.M., Srivastava V.M. (2022). Device modeling of organic photovoltaic cells with traditional and inverted cells using s-SWCNT:C60 as active layer. Nanomaterials.

[30] Moorthy V.M., Srivastava V.M. (2022). Effect of active layer thickness on organic thin-film transistors. ECS Trans..

[31] Bareket L., Waiskopf N., Rand D., Lubin G., David-Pur M., Ben-Dov J., Roy S., Eleftheriou C., Sernagor E., Cheshnovsky O. (2014). Semiconductor Nanorod—Carbon Nanotube Biomimetic Films for Wire-Free Photostimulation of Blind Retinas. Nano Lett..

[32] Antognazza M.R., Ghezzi D., Musitelli D., Garbugli M., Lanzani G. (2009). A hybrid solid-liquid polymer photodiode for the bioenvironment A hybrid solid-liquid polymer photodiode for the bioenvironment. Appl. Phys. Lett..

[33] Ghezzi D., Antognazza M.R., Maschio M.D., Lanzarini E., Benfenati F., Lanzani G. (2011). A hybrid bioorganic interface for neuronal photoactivation. Nat. Commun..

[34] Maya-Vetencourt J.F., Ghezzi D., Antognazza M.R., Colombo E., Mete M., Feyen P., Desii A., Buschiazzo A., Di Paolo M., Di Marco S. (2017). A fully organic retinal prosthesis restores vision in a rat model of degenerative blindness. Nat. Mater..

[35] Ghezzi D., Antognazza M.R., Maccarone R., Bellani S., Lanzarini E., Martino N., Mete M., Pertile G., Bisti S., Lanzani G. (2013). A polymer optoelectronic interface restores light sensitivity in blind rat retinas. Nat. Photon-.

[36] Antognazza M.R., Di Paolo M., Ghezzi D., Mete M., Di Marco S., Maya-Vetencourt J.F., Maccarone R., Desii A., Di Fonzo F., Bramini M. (2016). Characterization of a Polymer-Based, Fully Organic Prosthesis for Implantation into the Subretinal Space of the Rat. Adv. Heal. Mater..

[37] Narayan K.S., Gautam V., Bag M. (2015). Artificial Retina Device. U.S. Patent.

[38] Rathbun S.D. (2014). Chapter 1 Restoring Vision to the Blind: The New Age of Implanted Visual Prostheses Recent Advances in Retinal Stimulation: Clinical Applications. Transl. Vis. Sci..

[39] Lee D.Y., Lorach H., Huie P., Palanker D. (2016). Implantation of Modular Photovoltaic Subretinal Prosthesis. Ophthalmic Surg. Lasers Imaging Retin..

[40] Becerril H.A., Mao J., Liu Z., Stoltenberg R.M., Bao Z., Chen Y. (2008). Evaluation of Solution-Processed Reduced Graphene Oxide Films as Transparent Conductors. ACS Nano.

[41] Srivastava V.M., Singh G. (2014). MOSFET Technologies for Double-Pole Four Throw Radio Frequency Switch.

[42] Rathore R.S., Rana A.K., Srivastava V.M. Impact of oxide thickness fluctuation for resist- and spacer-defined FinFETs. Proceedings of the Annual 4th IEEE Latin American Electron Devices Conference (LAEDC).

[43] Li C., Chen Y., Wang Y., Iqbal Z., Chhowalla M., Mitra S. (2007). A fullerene—Single wall carbon nanotube complex for polymer bulk heterojunction photovoltaic cells. J. Mater. Chem..

[44] Bindl D.J., Wu M.-Y., Prehn F.C., Arnold M.S. (2010). Efficiently harvesting excitons from electronic type-controlled semiconducting carbon nanotube films. Nano Lett..

[45] Gong M., Shastry T.A., Xie Y., Bernardi M., Jasion D., Luck K.A., Marks T.J., Grossman J.C., Ren S., Hersam M.C. (2014). Polychiral Semiconducting Carbon Nanotube—Fullerene Solar Cells. Nano Lett..

[46] Swapna P., Rao Y.S. (2015). Fabrication and characterization of semiconducting single walled carbon nano tube based bulk hetero junction organic solar cell using spin coating technique. J. Chin. Adv. Mater. Soc..

[47] Moorthy V.M., Srivastava V.M. (2022). Device modelling and optimization of nanomaterial-based planar heterojunction solar cell (by varying the device dimensions and material parameters). Nanomaterials.

[48] Gowthaman N., Srivastava V.M. (2022). Mathematical modeling of drain current estimation in a CSDG MOSFET, based on La2O3 oxide layer with fabrication—A nanomaterial approach. Nanomaterials.

[49] Mutepfe C.D.K., Srivastava V.M. (2022). Design and implementation of graphene-based tunable microwave filter for THz applications. Nanomaterials.

[50] Meo M., Rossi M. (2006). Prediction of Young’s modulus of single wall carbon nanotubes by molecular-mechanics based finite element modelling. Compos. Sci. Technol..

[51] Park S.H., Roy A., Beaupré S., Cho S., E Coates N., Moon J.S., Moses D., Leclerc M., Lee K., Heeger A.J. (2009). Bulk heterojunction solar cells with internal quantum efficiency approaching 100%. Nat. Photon..

[52] Gerasimenko A.Y., Ten G.N., Ryabkin D.I., Shcherbakova N.E., Morozova E.A., Ichkitidze L.P. (2019). The study of the interaction mechanism between bovine serum albumin and single-walled carbon nanotubes depending on their diameter and concentration in solid nanocomposites by vibrational spectroscopy. Acta Part A Mol. Biomol. Spectrosc..

[53] Yu H., Li Y., Dong Y., Huang X. (2016). Fabrication and Optimization of Polymer Solar Cells Based on P3HT:PC_70_BM System. Int. J. Photoenergy.

[54] Pesonen M., Majumdar H.S., Kauppila J., Lukkari J., Österbacka R. (2012). Large-scale solution processable graphene-based thin film devices. MRS Proc..

[55] Pierre A., Lu S., Howard I.A., Facchetti A., Arias A.C. (2013). Empirically based device modeling of bulk heterojunction organic photovoltaics. J. Appl. Phys..

[56] Meyyappan M.V., Daniel R.J., Shanmugaraja P. Comparison studies of planar and bulk hetero junction nano photo diodes using carbon nano tube’s (CNT) and Graphene for Sub-retinal implant. Proceedings of the 2018 Conference on Emerging Devices and Smart Systems (ICEDSS).

[57] Ismail Y.A., Soga T., Jimbo T. (2015). Effect of Composition on Conjugation Structure and Energy Gap of P3HT:PCBM Organic Solar Cell. Int. J. N. Hor. Phys..

[58] Zhi-Hui F., Yan-Bing H., Quan-Min S., Li-Fang Q., Yan L., Lei Z., Xiao-Jun L., Feng T., Yong-Sheng W., Rui-Dong X. (2010). Polymer solar cells based on a PEDOT: PSS layer spin-coated under the action of an electric field. Chin. Phys. B.

[59] Li G., Shrotriya V., Huang J., Yao Y., Moriarty T., Emery K., Yang Y. (2005). High-efficiency solution processable polymer photovoltaic cells by self-organization of polymer blends. Nat. Mater..

[60] Das S., Alford T.L. (2014). Improved efficiency of P3HT: PCBM solar cells by incorporation of silver oxide interfacial layer. J. Appl. Phys..

[61] Otieno F., Shumbula N.P., Airo M., Mbuso M., Moloto N., Erasmus R.M., Quandt A., Wamwangi D. (2017). Improved efficiency of organic solar cells using Au NPs incorporated into PEDOT: PSS buffer layer. AIP Adv..

[62] Girtan M., Rusu M. (2010). Role of ITO and PEDOT: PSS in stability/degradation of polymer:fullerene bulk heterojunctions solar cells. Sol. Energy Mater. Sol. Cells.

[63] Keyvani-Someh E., Hennighausen Z., Lee W., Igwe R.C.K., Kramdi M.E., Kar S., Fenniri H. (2017). Organic Photovoltaics with Stacked Graphene Anodes. ACS Appl. Energy Mater..

[64] Wang Z., Puls C.P., Staley N.E., Zhang Y., Todd A., Xu J., Howsare C.A., Hollander M.J., Robinson J.A., Liu Y. (2011). Technology ready use of single layer graphene as a transparent electrode for hybrid photovoltaic devices. Phys. E Low-Dimens. Syst. Nanostructures.

[65] Moorthy V.M., Sugantharathnam M., Rathnasami J.D., Pattan S. (2019). Design and characterization of graphene-based nano-photodiode array device for photo-stimulation of subretinal implant. Micro Nano Lett..

[66] Tung V.C., Chen L.-M., Allen M.J., Wassei J.K., Nelson K., Kaner R.B., Yang Y. (2009). Low-Temperature Solution Processing of Graphene- Carbon Nanotube Hybrid Materials for High-Performance Transparent Conductors. Nano Lett..

[67] Yin Z., Sun S., Salim T., Wu S., Huang X., He Q., Lam Y.M., Zhang H. (2010). Organic Photovoltaic Devices Using Highly Flexible Reduced Graphene Oxide Films as Transparent Electrodes. ACS Nano.

[68] Khan S.M., Hasan A.A., Saha A. (2015). Photovoltaic Performance of P3HT:SWCNT:PCBM based Bulk Heterojunction Solar Cell. Ph.D. Thesis.

